# Rhinitis, sinusitis and ocular disease – 2087. Usefulness of impulse oscilometry and fractional exhaled nitric oxide in children with eosinophilic bronchitis

**DOI:** 10.1186/1939-4551-6-S1-P166

**Published:** 2013-04-23

**Authors:** Yoon-Hee Kim, Kyung-Won Kim, Myung-Hyun Sohn, Kyu-Earn Kim

**Affiliations:** 1 Pediatrics, Yonsei University College of Medicine, Seoul, South Korea

## Background

Eosinophilic bronchitis (EB) is a common cause of chronic cough. Although EB shares many immunopathologic features with asthma, it does not show airway hyperresponsiveness or reversible airway obstruction by spirometry. Compared to healthy children without pulmonary disease, we hypothesized that EB patients would demonstrate abnormal pulmonary function and inflammation with impulse oscillometry (IOS) and fractional exhaled nitric oxide (FeNO), which are more sensitive tests of these parameters than spirometry.

## Methods

A total of 232 children with asthma, 109 with EB, and 115 control subjects were enrolled. We compared pulmonary function parameters and FeNO levels among the three groups. Additionally, we designated a screening cutoff value of FeNO combined with IOS parameters to distinguish EB from the control group, and identify which children with EB have more asthmatic characteristics.

## Results

By IOS, the bronchodilator response of the EB and asthma groups increased significantly compared to controls for both reactance at 5 Hz (Δ X5) and reactance area (Δ AX) (*P* < 0.0001). Cutoff values to distinguish EB from controls were a Δ X5 of -20% (sensitivity, 77.5%; specificity, 49.6%), and Δ AX of -30% (sensitivity, 75.0%; specificity, 46.0%), when the FeNO is 20 ppb.

## Conclusions

Reversible airway obstruction in IOS and elevated FeNO levels can be detected in children with EB. This would support that EB in children shows airway characteristics similar to those of asthma, and that a continuum exists between asthma and EB.

